# Expression of α-synuclein is regulated in a neuronal cell type-dependent manner

**DOI:** 10.1007/s12565-018-0464-8

**Published:** 2018-10-25

**Authors:** Katsutoshi Taguchi, Yoshihisa Watanabe, Atsushi Tsujimura, Masaki Tanaka

**Affiliations:** 1 Department of Anatomy and Neurobiology, Graduate School of Medical Science, Kyoto Prefectural University of Medicine, Kawaramachi-Hirokoji, Kamikyo-ku, Kyoto, 602-8566 Japan; 2 Department of Basic Geriatrics, Graduate School of Medical Science, Kyoto Prefectural University of Medicine, Kawaramachi-Hirokoji, Kamikyo-ku, Kyoto, 602-8566 Japan

**Keywords:** Dementia with Lewy bodies, Excitatory neuron, Inhibitory neuron, Parkinson’s disease, Synapse

## Abstract

α-Synuclein, the major component of Lewy bodies (LBs) and Lewy neurites (LNs), is expressed in presynapses under physiologically normal conditions and is involved in synaptic function. Abnormal intracellular aggregates of misfolded α-synuclein such as LBs and LNs are pathological hallmarks of synucleinopathies, including Parkinson’s disease (PD) and dementia with Lewy bodies (DLB). According to previous studies using pathological models overexpressing α-synuclein, high expression of this protein in neurons is a critical risk factor for neurodegeneration. Therefore, it is important to know the endogenous expression levels of α-synuclein in each neuronal cell type. We previously reported differential expression profiles of α-synuclein in vitro and in vivo. In the wild-type mouse brain, particularly in vulnerable regions affected during the progression of idiopathic PD, α-synuclein is highly expressed in neuronal cell bodies of some early PD-affected regions, such as the olfactory bulb, the dorsal motor nucleus of the vagus, and the substantia nigra pars compacta. Synaptic expression of α-synuclein is mostly accompanied by expression of vesicular glutamate transporter-1, an excitatory synapse marker protein. In contrast, α-synuclein expression in inhibitory synapses differs among brain regions. Recently accumulated evidence indicates the close relationship between differential expression profiles of α-synuclein and selective vulnerability of certain neuronal populations. Further studies on the regulation of α-synuclein expression will help to understand the mechanism of LB pathology and provide an innovative therapeutic strategy to prevent PD and DLB onset.

## Introduction

α-Synuclein is a major constituent of Lewy bodies (LBs) and Lewy neurites (LNs), which are pathological hallmarks of synucleinopathies, including Parkinson’s disease (PD) and dementia with Lewy bodies (DLB) (Spillantini et al. 1998; Dickson 2001; Stefanis 2012). Several missense mutations, as well as duplicate and triplicate regions of the α-synuclein gene are responsible for familial PD (Polymeropoulos et al. 1997; Zarranz et al. 2004; Kruger et al. 1998; Singleton et al. 2003; Chartier-Harlin et al. 2004). In studies of α-synuclein pathogenicity, it was demonstrated that overexpression of α-synuclein in neurons results in the formation of inclusion bodies and neuronal loss (Masliah et al. 2000; Van der Perren et al. 2015; Singleton et al. 2003). Therefore, an increase in the intracellular amount of α-synuclein is a probable risk factor for neurodegeneration.

Expression of α-synuclein is regulated by various transcription factors, such as zinc finger and SCAN domain containing 21 (ZSCAN21) (Clough et al. 2009; Dermentzaki et al. 2016), GATA-1 and GATA-2 (Scherzer et al. 2008), Nurr1 (Yang and Latchman 2008), TRIM32 (Pavlou et al. 2017), and p27^Kip1^ (Gallastegui et al. 2018). These transcription factors interact directly with the promotor region of α-synuclein, and this critical link between these transcription factors and α-synuclein may enable the design of therapies to lower production of α-synuclein. Further studies of the regulation machinery of α-synuclein expression via these transcription factors will help develop novel therapeutic strategies for synucleinopathies.

According to a recent study, β2-adrenoreceptor (β2AR) was identified as a novel regulator of the α-synuclein gene (Mittal et al. 2017). β2AR activation by selective agonists reduces α-synuclein expression in mouse substantia nigra. Conversely, suppression of β2AR expression or chemical inhibition of β2AR activity increases α-synuclein expression. Furthermore, longitudinal studies of incident PD throughout Norway showed that the β2AR antagonist, propranolol, is associated with a markedly increased risk of PD. However, salbutamol, a β2AR agonist, is associated with a decreased risk of PD. Thus, fine-tuning of intrinsic α-synuclein expression levels could constitute an innovative therapeutic strategy to prevent PD onset.

Expression levels and subcellular distribution of α-synuclein in each neuronal cell type are related closely to the pathogenicity and the physiological function of α-synuclein. In this review, we focus on the characteristic profile of α-synuclein expression in vitro and in vivo, and further discuss new findings obtained from recent studies on this protein.

### Differential expression of α-synuclein under physiological conditions

α-Synuclein is enriched in brain and is localized at presynapses under physiologically normal conditions in vitro and in vivo (Withers et al. 1997; Totterdell et al. 2004; Totterdell and Meredith 2005; Vivacqua et al. 2011). It has been suggested that α-synuclein plays a role in the generation and maintenance of synapses because this protein appears earlier than synaptophysin—a synaptic vesicle protein—during development of the central nervous system (CNS) and is localized to axon terminals throughout the adult mammalian brain (Hsu et al. 1998; Petersen et al. 1999).

α-Synuclein binds directly to synaptobrevin-2 in presynaptic regions and functions to sustain soluble *N*-ethylmaleimide-sensitive factor attachment protein receptor (SNARE)-complex assembly in vivo and in vitro (Burre et al. 2010). According to a more recent study, α-synuclein modulates the kinetics of exocytotic events, promoting cargo discharge and reducing pore closure (Logan et al. 2017). Thus, these studies indicate a synaptic function of α-synuclein during the neurotransmission process.

It is believed that α-synuclein is generally expressed in synapses, whereas the amount of α-synuclein protein expressed in each cell differs between different neuronal cell types. Li and colleagues indicated that α-synuclein is present abundantly in central catecholaminergic systems (Li et al. 2002), whereas α-synuclein expression is comparatively weak in many cholinergic brain regions.

**Fig. 1:**
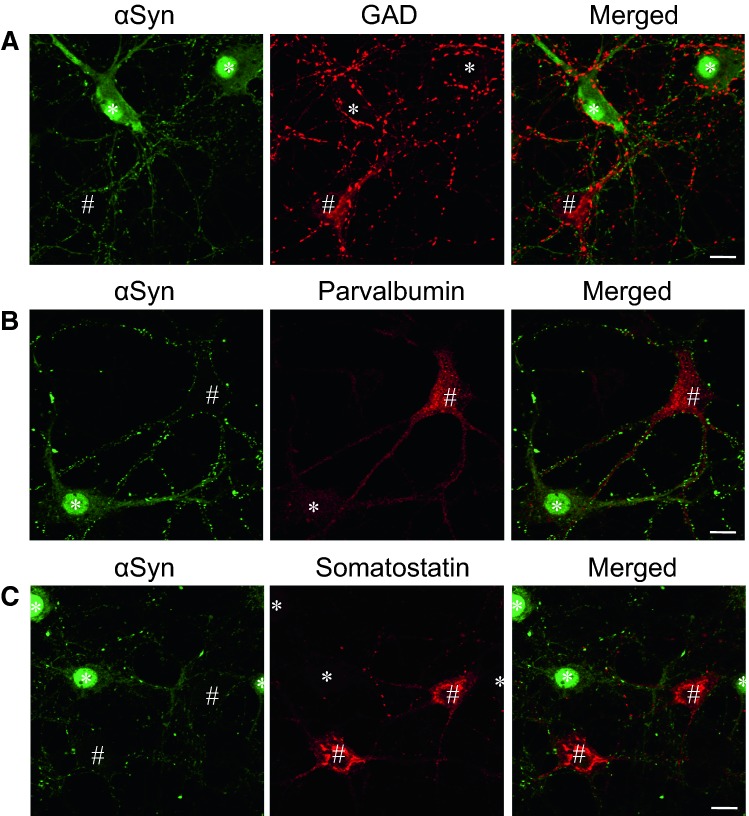
**a**–**c** Low expression of α-synuclein (*αSyn*) in hippocampal inhibitory neurons. # Cells expressing the inhibitory neuronal marker proteins, glutamic acid decarboxylase (*GAD*) (**a**), parvalbumin (**b**), and somatostatin (**c**), show low expression of αSyn. Cells with high expression of αSyn are labeled with* asterisks*.* Bars* 10 µm (Taguchi et al. 2014)

**Fig. 2:**
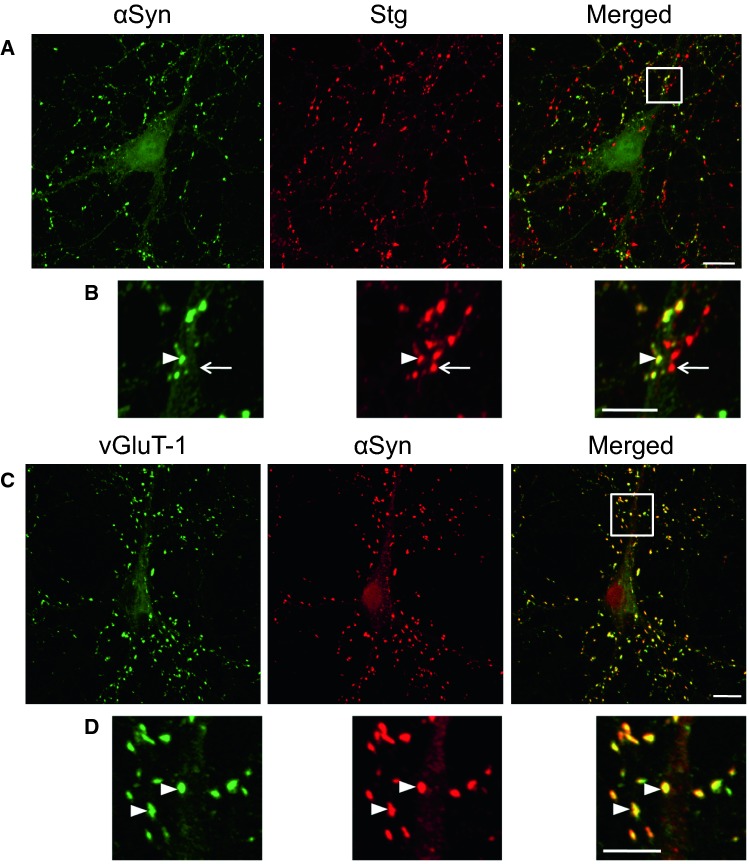
Presynaptic localization of α-synuclein in hippocampal excitatory neurons. **a**, **b** Confocal images of double immunostaining for α-synuclein (αSyn) and synaptotagmin (Stg). The region marked by a* white square* in **a** is magnified in **b**.* Arrowhead* in **b** indicates the presynapse, expressing both αSyn and Stg. However, there are some Stg-positive synapses lacking αSyn (*arrow*). **c**, **d** Confocal images of double immunostaining for αSyn and vGluT-1. The region marked by a* white square* in **c** is magnified in **d**. αSyn is clearly colocalized with vGluT-1 in **d** (*arrowheads*).* Bars***a**, **c** 10 µm; **c**, **d** 5 µm (Taguchi et al. 2014)

We previously investigated the subcellular distribution of α-synuclein in normal and pathological conditions using primary cultured hippocampal neurons (Taguchi et al. 2014). While some neurons expressed high levels of α-synuclein in presynapses expressing synaptotagmin (Stg)—a presynaptic marker protein—and cell bodies, other neurons either did not express the protein, or only very weakly (Figs. 1, 2a,b). These α-synuclein-negative cells were identified as inhibitory neurons expressing glutamic acid decarboxylase (GAD) (Fig. 1a), parvalbumin (Fig. 1b), or somatostatin (Fig. 1c). In contrast, α-synuclein-positive synapses were clearly colocalized with vesicular glutamate transporter-1 (vGluT-1)—an excitatory synapse marker protein (Fig. 2c,d). This characteristic expression pattern of α-synuclein is conserved in the hippocampus in vivo.

According to a previous study, α-synuclein plays a role in maintaining the overall size of the recycling pool of synaptic vesicles (Scott and Roy 2012). The sizes of both the recycling pool and total vesicular pool are more variable at glutamatergic synapses than at gamma-aminobutyric acid (GABAergic) synapses (Moulder et al. 2007). This heterogeneity of the size of the recycling pool at glutamatergic synapses may provide a dynamic range for synaptic strength that is not present at GABAergic synapses (Moulder and Mennerick 2005; Moulder et al. 2007). Therefore, α-synuclein might act as a modulator of the size of the recycling pool at excitatory synapses.

### Cell-type dependent aggregate formation and synapse impairment

**Fig. 3a–c:**
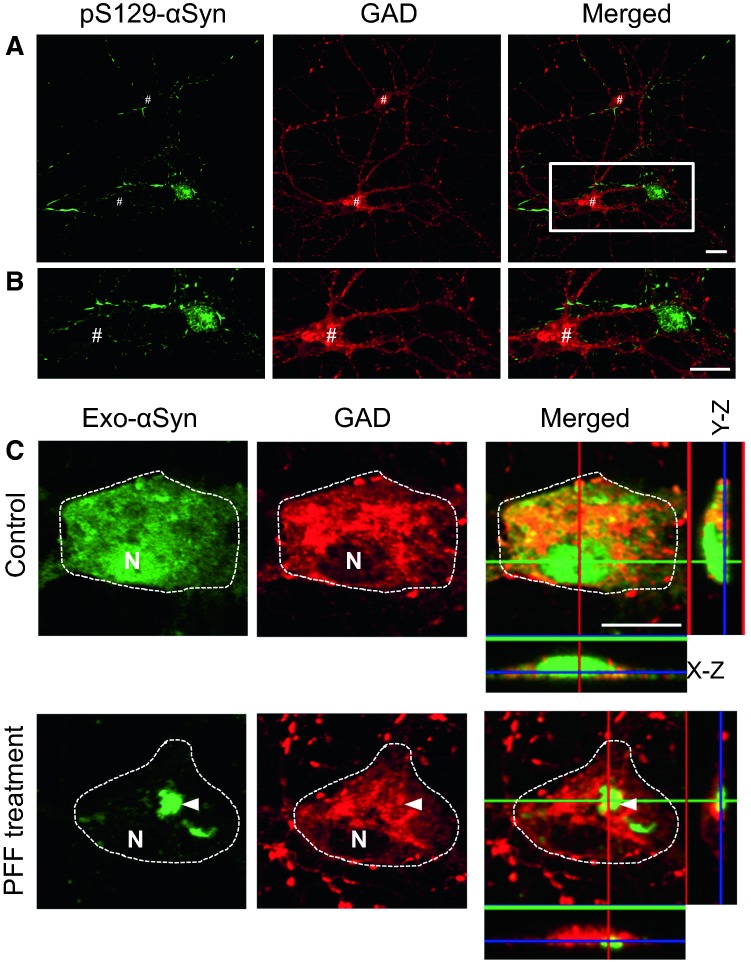
Formation of intracellular aggregates of α-synuclein. **a**, **b** Confocal images of double immunostaining for phosphorylated α-synuclein (pS129-αSyn) and GAD after treatment with preformed fibrils (PFFs) of α-synuclein. The region marked by a* white square* in **a** is magnified in **b**. Immunoreactivity of pS129-αSyn is observed as intracellular fibrous aggregates or inclusion bodies. GAD-positive neurons indicated by* #* are free of α-synuclein aggregate formation. GAD signals are not colocalized with pS129-αSyn. **c** In the absence of PFF treatment, exogenous human α-synuclein (Exo-αSyn) was distributed diffusely in the cell body of GAD neurons (*control*). After PFF treatment, intracellular inclusions positive for αSyn are induced in the GAD-positive cells expressing Exo-αSyn. Cell bodies are enclosed by* white dotted lines*. *N* indicates the location of the nucleus.* Bars* 10 µm (Taguchi et al. 2014)

Intracellular aggregates such as LBs and LNs are composed mainly of α-synuclein. These aggregates are formed by recruitment of the intrinsic soluble α-synuclein into the insoluble aggregate core. Therefore, endogenous expression of α-synuclein is required for aggregate formation (Volpicelli-Daley et al. 2011). Preformed fibrils (PFFs) prepared from recombinant α-synuclein can induce the formation of LB-like aggregates. If inhibitory hippocampal neurons have low expression levels of endogenous α-synuclein as described above, they may not be able to form LB-like aggregates. As expected, most of the GAD-positive inhibitory hippocampal neurons were free of LB-like aggregates (Fig. 3a,b) (Taguchi et al. 2014). However, LB-like aggregate formation was induced successfully by PFFs in hippocampal inhibitory neurons overexpressing exogenous human α-synuclein (Fig. 3c). The low frequency of LB-like aggregate appearance is probably due to the lower amount of endogenous α-synuclein expressed in the inhibitory neurons. As described below, inhibitory neurons in the cerebral cortex and some other regions including the hippocampus show low expression of α-synuclein in adult mouse brain (Taguchi et al. 2016). A pathological study of DLB patients reported that parvalbumin-containing cortical neurons are free of LBs and spared from neurodegeneration, although the basal expression level of α-synuclein was not determined (Gomez-Tortosa et al. 2001). Formation of LBs composed of α-synuclein might be related to the endogenous expression levels of α-synuclein, which is regulated in a neuronal cell type-dependent manner.

Presynaptic α-synuclein aggregates in the cortex of DLB brain correlate with reduced dendritic spines, suggesting that these aggregates contribute to synapse loss and cognitive dysfunction (Kramer and Schulz-Schaeffer 2007). It was recently demonstrated that exposure of wild-type neurons to PFFs causes a significant reduction in mushroom-like stable spine density (Froula et al. 2018). Interestingly, this reduction of spine density is observed only in wild-type neurons expressing endogenous α-synuclein, but not in α-synuclein knockout neurons. The authors hypothesized that these changes in spine morphology result from PFF-induced corruption of endogenous α-synuclein expressed in the hippocampal neurons. This latter study focused on the morphology and function of glutamatergic excitatory synapses at early pathological stages before neuronal cell death induced by PFF-treatment, and further indicated the reduced frequency and amplitudes of spontaneous Ca^2+^ transients. Thus, endogenous expression levels of α-synuclein might be a critical factor for synapse impairment at early pathological stages during the progression of neurodegeneration.

### Brain region-dependent differential expression of α-synuclein

In the pathological brain, Braak and colleagues proposed a caudorostral process associated with sporadic PD progression from the lower brain stem through the basal midbrain and forebrain into the cerebral cortex (Braak and Del Tredici 2009; Braak et al. 2003). Their studies indicated specifically affected brain regions, such as the olfactory bulb, dorsal motor nucleus of the vagus (DMN), and substantia nigra at early stages of PD, and also the amygdala, hippocampus, and neocortex at later stages. As discussed above, endogenous α-synuclein expression is required for LB-like aggregate formation (Volpicelli-Daley et al. 2011; Taguchi et al. 2014). Therefore, we further investigated the precise expression profile of α-synuclein in the wild-type adult mouse brain, particularly in the vulnerable regions affected during the progression of idiopathic PD (Taguchi et al. 2016).

**Fig. 4a,b:**
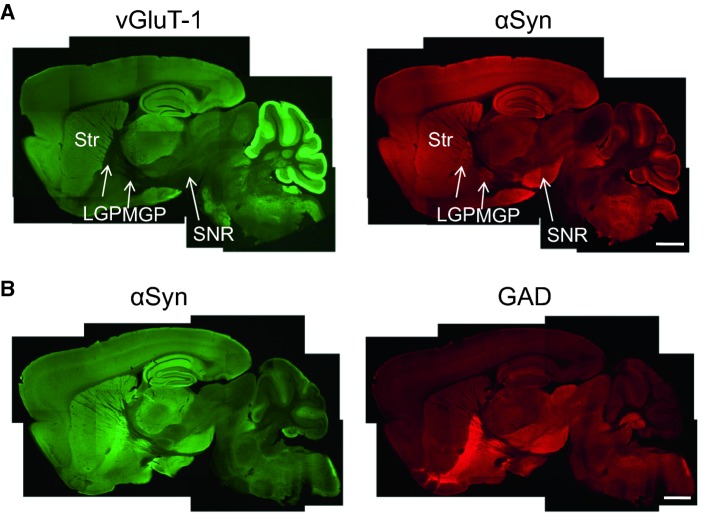
Sagittal plane brain distribution of α-synuclein compared with vGluT-1 or GAD. **a** There is a similar distribution pattern between α-synuclein (αSyn) and vGluT-1 except for some regions, including the lateral globus pallidus (LGP), medial globus pallidus (MGP), and substantia nigra pars reticulata (SNR). In these regions, αSyn is colocalized with GAD (**b**). *Str* striatum.* Bars* 1 mm (Taguchi et al. 2016)

α-Synuclein is broadly expressed in the mouse brain (Fig. 4). There is a similar distribution pattern between vGluT-1 and α-synuclein, except for the lateral and medial globus pallidus (LGP and MGP) and substantia nigra pars reticulata (SNR) (Fig. 4a). In contrast, GAD shows a complementary weak expression in the cerebral cortex, hippocampus, thalamus, and striatum (Str), but shows a strong expression in the LGP, MGP, and SNR, where vGluT-1 expression is very weak (Fig. 4b).

**Fig. 5a–d:**
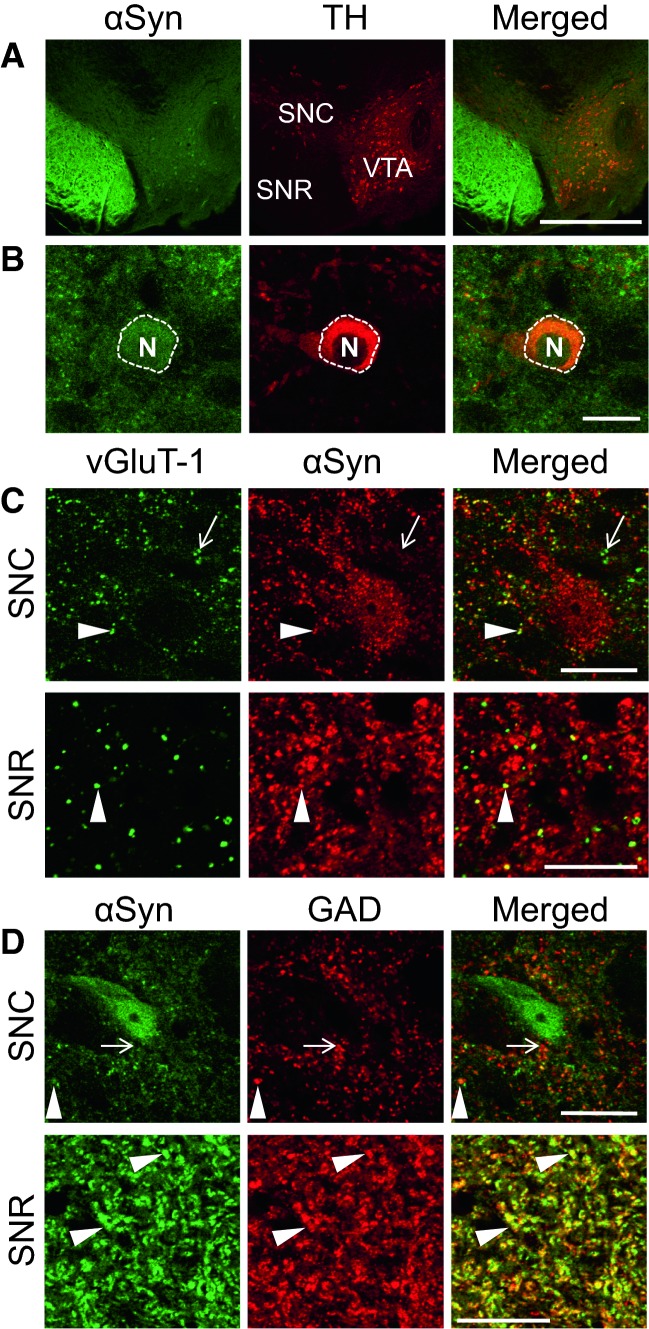
Localization of α-synuclein in the substantia nigra. **a** Tyrosine hydroxylase (*TH*)-positive neurons are distributed in the substantia nigra pars compacta (*SNC*) and the ventral tegmental area (*VTA*). These cells are *αSyn*-positive. **b** In TH-positive neurons located in the SNC, αSyn is enriched within the cell body. **c** αSyn is partially colocalized with vGluT-1 in the SNC and the substantia nigra pars reticulata (*SNR*). Although vGluT-1-positive synapses without the expression of αSyn are also observed in the SNC (*arrow*), scattered punctate signals of vGluT-1 in the SNR are clearly colocalized with αSyn (*arrowhead*). **d** αSyn is partially colocalized with GAD in the SNC (*arrowhead*). In the SNR, αSyn is clearly colocalized with GAD. *N* nucleus.* Bars***a** 500 µm, **b**–**d** 20 µm (Taguchi et al. 2016)

**Fig. 6a–d:**
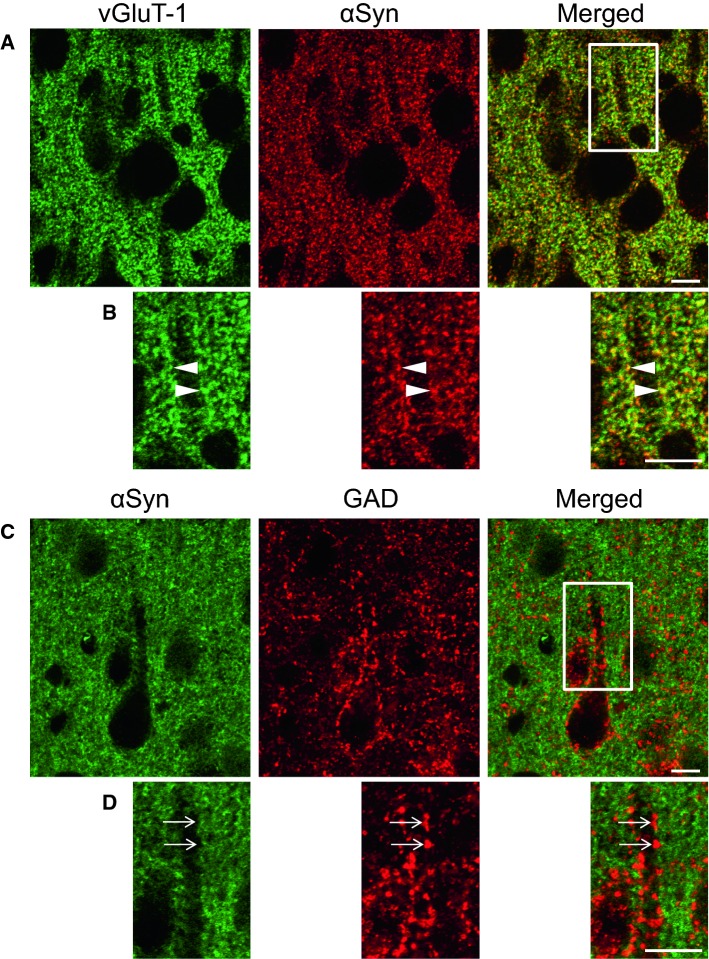
Localization of α-synuclein in the cerebral cortex. **a**, **b** αSyn is clearly colocalized with vGluT-1 (* arrowheads*). **c**, **d** αSyn is not colocalized with GAD (* arrows*). The regions marked by* white squares* in **a** and **c** are magnified in **b** and **d**, respectively.* Bars* 10 µm (Taguchi et al. 2016)

As summarized in Table 1, α-synuclein is highly expressed in the neuronal cell bodies of some early PD-affected regions, such as the olfactory bulb, DMN, substantia nigra pars compacta (SNC; Fig. 5a, b), and lateral and medial mammillary nucleus (LMN and MMN). Synaptic expression of α-synuclein is mostly accompanied by the expression of vGluT-1. In contrast, expression profiles of α-synuclein in inhibitory synapses are different among brain regions. α-Synuclein is clearly expressed in GAD-positive inhibitory synapses in the external plexiform layer of the olfactory bulb, LGP, MGP, and SNR, but not in the cerebral cortex, hippocampus, subthalamic nucleus, or thalamus. In the SNC, α-synuclein partially colocalizes with vGluT-1; there are α-synuclein-positive and -negative excitatory synapses (Fig. 5c). Regarding GAD expression, there are also α-synuclein-positive and -negative inhibitory synapses (Fig. 5d). In this region, therefore, α-synuclein-positive synapses comprise both excitatory and inhibitory synapses. In the SNR, dense α-synuclein and GAD-immunoreactive terminals are clearly colocalized, whereas excitatory terminals are only sparsely distributed (Fig. 5c,d). Although α-synuclein is partially colocalized with vGluT-1, those excitatory terminals co-express α-synuclein. Therefore, in the SNR, α-synuclein is expressed mainly in inhibitory synapses. Conversely, in the cerebral cortex, α-synuclein is clearly expressed in excitatory synapses (Fig. 6a, b), but not in inhibitory synapses (Fig. 6c, d).

**Table 1:** Expression profile of α-synuclein in wild-type mouse brain. *GL* Glomerular layer, *EPL* external plexiform layer, *IML* inframitral layer, *Ant* anterior, *N* nucleus, *DMN* dorsal motor nucleus of the vagus, *GRN* gigantocellular reticular nucleus, *SNC* substantia nigra pars compacta, *SNR* substantia nigra pars reticulata, *GP* globus pallidus, *LMN* lateral mammillary nucleus, *MMN* medial mammillary nucleus This table was modified from Taguchi et al. (2016)

	Presynaptic IR^a^	Somatic IR^b^	vGluT-1/αSyn^b^	GAD/αSyn^b^
Olfactory bulb^c^				
GL	++	++	+	+
EPL	+++	(−)	(−)	++
IML	++	(−)	++	(−)
Ant olfactory N	++	(−)	++	(−)
Medulla oblongata				
DMN^c^	++	++	+	+
GRN^c^	+	(−)	+	+
Raphe pallidus N^c^	+	(−)	+	+
Raphe obscurus N^c^	+	(−)	+	+
Inferior olive	+	(−)	+	+
Pons				
Locus coeruleus^c^	++	(−)	+	+
Parabrachial N^c^	+++	(−)	+	+
Midbrain				
SNC^c^	+	++	+	+
SNR	+++	(−)	+	++
Cerebrum				
Amygdala				
Central^c^	+++	(−)	+	+
Basolateral^c^	++	(−)	++	+
Cerebral cortex	++	(−)	++	(−)
Piriform cortex^c^	++	(−)	++	+
Entorhinal cortex	++	(−)	++	+
Hippocampus (CA1)	+++	(−)	++	(−)
Striatum	++	(**−**)	++	(−)
Lateral GP	+++	(−)	+	++
Medial GP	+++	(−)	+	++
Thalamus	++	(−)	++	(−)
Subthalamic N	+	(−)	++	(−)
LMN/MMN	++	++	+	+
Cerebellum				
Molecular layer	+++	(−)	+	(−)
Granule cell layer	+	(−)	++	(−)

^a^Intensity of α-synuclein immunoreactivity graded as absent (−), weak (+), moderate (++), and strong (+++)

^b^++: clearly colocalized or positive, +: partially colocalized, (−): not colocalized or negative

^c^Early-affected PD brain regions

Collectively, some neurons in the early PD-affected regions express high levels of perikaryal α-synuclein, as happens in the mouse brain (Table 1). Synaptic expression of α-synuclein is different in various brain regions. Interestingly, early PD-affected regions tend to have both excitatory and inhibitory synapses expressing α-synuclein, as indicated by superscript c in Table 1. However, α-synuclein is expressed only in excitatory synapses of the affected regions, such as the cerebral cortex, hippocampus, thalamus, and subthalamic nucleus at later PD stages, although there are also inhibitory synapses in those regions. Because α-synuclein is involved in synaptic function, expression of α-synuclein in inhibitory synapses of the early PD-affected regions might influence the local milieu and the fragility of vulnerable neurons expressing high levels of α-synuclein over time.

**Fig. 7:**
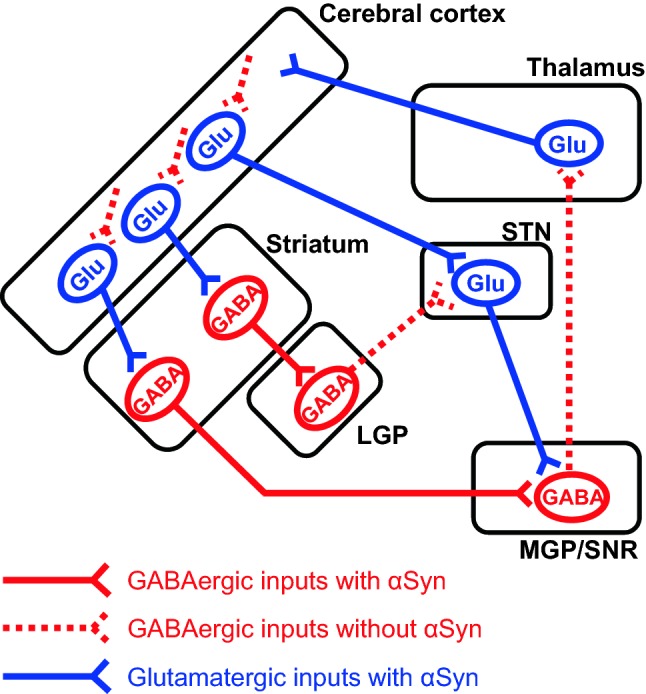
Expression profile of α-synuclein in the basal ganglia circuit. αSyn-positive inhibitory afferents are located in the lateral globus pallidus (*LGP*), medial globus pallidus (*MGP*), and substantia nigra pars reticulata (*SNR*). These regions receive the inhibitory inputs from striatal GABAergic (medium spiny) neurons (Taguchi et al. 2016)

In the LGP, MGP and SNR, α-synuclein is clearly expressed in inhibitory synapses. These regions receive GABAergic afferents from striatal medium spiny neurons (MSNs) (Smith and Bolam 1990; Utter and Basso 2008). Therefore, it is suggested that striatal MSNs express α-synuclein (Fig. 7). However, other inhibitory neurons located in the LGP, MGP, and SNR project their axons to the thalamus and subthalamic nuclei, but they do not express α-synuclein. This difference might be a result of the unique neurochemical and electrophysiological properties of MSNs. Besides GABA, MSNs express substance P or enkephalin-like peptides as their neurotransmitters (Smith and Bolam 1990; McGinty 2007; Utter and Basso 2008) and fire at low rates in irregular bursts (Wilson 1993). In addition, MSN axons are unmyelinated (Miyazaki et al. 2014). Braak and Del Tredici described that neurons prone to LB pathology are projection neurons with thin, long axons that are unmyelinated or only sparsely myelinated (Braak and Del Tredici 2004, 2009). Actually, LBs are found in MSNs at stage III of the PD brain (Mori et al. 2002). In the cerebral and cerebellar cortices, GABAergic interneurons with short axons do not express α-synuclein (Taguchi et al. 2016). These results suggest that neuronal expression of α-synuclein is dependent on the morphological characteristics of axons, such as whether they are myelinated or whether they are long or short.

### Selective vulnerability of α-synuclein-enriched neurons

Recently, it was reported that differential α-synuclein expression contributes to selective vulnerability of hippocampal neuron subpopulations to fibril-induced toxicity (Luna et al. 2018). Previously, Luk and colleagues reported that, in non-transgenic wild-type mice, a single intrastriatal inoculation of PFFs of α-synuclein leads to the propagation of LB-like pathology in anatomically interconnected brain regions (Luk et al. 2012). Using this experimental model, they demonstrated that Math2-expressing hippocampal *Cornu ammonis* (CA) neurons are highly susceptible to pathological seeding with the PFFs, in contrast to the dentate gyrus (DG) neurons expressing Prox1 (Luna et al. 2018). Glutamatergic excitatory neurons derived from CA regions express the transcription factor Math2, whereas DG neurons express another transcription factor, Prox1 (Bagri et al. 2002; Sugiyama et al. 2014). The hippocampal CA is a region significantly affected by α-synuclein pathology in advanced PD and DLB compared with the DG (Armstrong et al. 2014; Hall et al. 2014). Interestingly, Math2-expressing CA neurons show higher levels of α-synuclein expression, whereas Prox1-expressing DG neurons express low levels of α-synuclein and are resistant relative to the Math2-expressing subpopulations (Luna et al. 2018). These results indicate that PFF-induced cell susceptibility is closely related to the endogenous expression levels of α-synuclein, which are regulated differentially in each neuronal cell-type.

In postmortem human brains, regional levels of physiological α-synuclein are directly associated with LB pathology (Erskine et al. 2018). Quantitative imaging and western blotting analysis demonstrated that brain regions less relevant to LB pathology, such as primary visual cortex and cerebellar cortex, show strikingly lower expression levels of α-synuclein. Recruitment of soluble α-synuclein into the intracellular aggregates is required for the process of pathological LB propagation in brain, as described above. Therefore, these results are consistent with previous studies using various in vitro and in vivo models. However, brain regions with the greatest proclivity to LB pathology did not have the highest levels of endogenous α-synuclein expression, and it was suggested that expression levels of α-synuclein are not the sole determinants of cell vulnerability. Vulnerability to LB pathology is the product of anatomical connectivity and region autonomous factors, with a baseline level of physiological α-synuclein expression necessary for pathology to develop (Erskine et al. 2018). For instance, higher α-synuclein expression is observed within the cell bodies of dopamine neurons in both the SNC and ventral tegmental area (VTA). However, dopaminergic neurons in the VTA are much less affected in PD. This difference might not be attributable to the expression levels of α-synuclein, however, because the difference in pacemaking mechanisms and engagement of L-type calcium channels in neurons of the SNC and VTA has been reported to cause a difference in their vulnerability (Guzman et al. 2010; Khaliq and Bean 2010).

## Conclusion

**Fig. 8:**
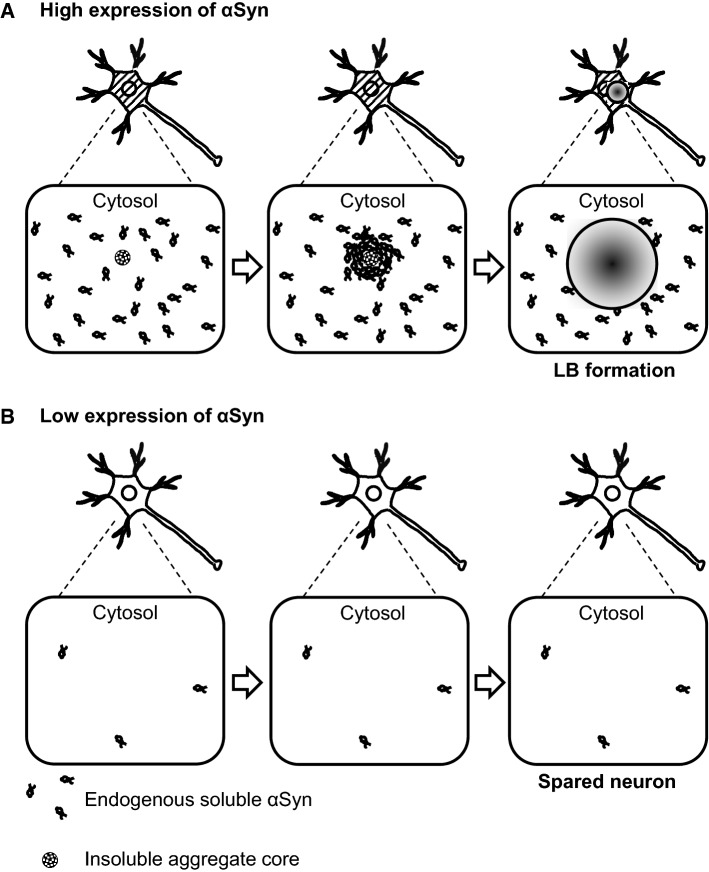
Models of the relationship between α-synuclein expression levels and Lewy body (LB) formation. **a** LB formed by recruitment of endogenous soluble αSyn into the insoluble aggregate core. **b** Neurons with low expression of αSyn are spared from LB pathology

High expression of α-synuclein is a critical risk factor for aggregate formation and neuronal loss (Fig. 8). Accumulated evidence suggests the close relationship between differential expression of α-synuclein and selective vulnerability of certain neuronal populations. Factors other than α-synuclein expression level may also be involved in vulnerability. Further investigation of the regulation of α-synuclein expression will help understand the mechanism of LB pathology and provide an innovative therapeutic strategy to prevent PD and DLB onset.

## References

[CR1] Armstrong RA, Kotzbauer PT, Perlmutter JS (2014). A quantitative study of alpha-synuclein pathology in fifteen cases of dementia associated with Parkinson disease. J Neural Transm (Vienna).

[CR2] Bagri A, Gurney T, He X (2002). The chemokine SDF1 regulates migration of dentate granule cells. Development.

[CR3] Braak H, Del Tredici K (2004). Poor and protracted myelination as a contributory factor to neurodegenerative disorders. Neurobiol Aging.

[CR4] Braak H, Del Tredici K (2009). Neuroanatomy and pathology of sporadic Parkinson’s disease. Adv Anat Embryol Cell Biol.

[CR5] Braak H, Rub U, Gai WP, Del Tredici K (2003). Idiopathic Parkinson’s disease: possible routes by which vulnerable neuronal types may be subject to neuroinvasion by an unknown pathogen. J Neural Transm (Vienna).

[CR6] Burre J, Sharma M, Tsetsenis T, Buchman V, Etherton MR, Sudhof TC (2010). Alpha-synuclein promotes SNARE-complex assembly in vivo and in vitro. Science.

[CR7] Chartier-Harlin MC, Kachergus J, Roumier C (2004). Alpha-synuclein locus duplication as a cause of familial Parkinson’s disease. Lancet.

[CR8] Clough RL, Dermentzaki G, Stefanis L (2009). Functional dissection of the alpha-synuclein promoter: transcriptional regulation by ZSCAN21 and ZNF219. J Neurochem.

[CR9] Dermentzaki G, Paschalidis N, Politis PK, Stefanis L (2016). Complex effects of the ZSCAN21 transcription factor on transcriptional regulation of alpha-synuclein in primary neuronal cultures and in vivo. J Biol Chem.

[CR10] Dickson DW (2001). Alpha-synuclein and the Lewy body disorders. Curr Opin Neurol.

[CR11] Erskine D, Patterson L, Alexandris A (2018). Regional levels of physiological alpha-synuclein are directly associated with Lewy body pathology. Acta Neuropathol.

[CR12] Froula JM, Henderson BW, Gonzalez JC (2018). Alpha-synuclein fibril-induced paradoxical structural and functional defects in hippocampal neurons. Acta Neuropathol Commun.

[CR13] Gallastegui E, Domuro C, Serratosa J (2018). p27^Kip1^ regulates alpha-synuclein expression. Oncotarget.

[CR14] Gomez-Tortosa E, Sanders JL, Newell K, Hyman BT (2001). Cortical neurons expressing calcium binding proteins are spared in dementia with Lewy bodies. Acta Neuropathol.

[CR15] Guzman JN, Sanchez-Padilla J, Wokosin D (2010). Oxidant stress evoked by pacemaking in dopaminergic neurons is attenuated by DJ-1. Nature.

[CR16] Hall H, Reyes S, Landeck N (2014). Hippocampal Lewy pathology and cholinergic dysfunction are associated with dementia in Parkinson’s disease. Brain.

[CR17] Hsu LJ, Mallory M, Xia Y (1998). Expression pattern of synucleins (non-Abeta component of Alzheimer’s disease amyloid precursor protein/alpha-synuclein) during murine brain development. J Neurochem.

[CR18] Khaliq ZM, Bean BP (2010). Pacemaking in dopaminergic ventral tegmental area neurons: depolarizing drive from background and voltage-dependent sodium conductances. J Neurosci.

[CR19] Kramer ML, Schulz-Schaeffer WJ (2007). Presynaptic alpha-synuclein aggregates, not Lewy bodies, cause neurodegeneration in dementia with Lewy bodies. J Neurosci.

[CR20] Kruger R, Kuhn W, Muller T (1998). Ala30Pro mutation in the gene encoding alpha-synuclein in Parkinson’s disease. Nat Genet.

[CR21] Li J, Henning Jensen P, Dahlstrom A (2002). Differential localization of alpha-, beta- and gamma-synucleins in the rat CNS. Neuroscience.

[CR22] Logan T, Bendor J, Toupin C, Thorn K, Edwards RH (2017). Alpha-synuclein promotes dilation of the exocytotic fusion pore. Nat Neurosci.

[CR23] Luk KC, Kehm V, Carroll J (2012). Pathological alpha-synuclein transmission initiates Parkinson-like neurodegeneration in nontransgenic mice. Science.

[CR24] Luna E, Decker SC, Riddle DM (2018). Differential alpha-synuclein expression contributes to selective vulnerability of hippocampal neuron subpopulations to fibril-induced toxicity. Acta Neuropathol.

[CR25] Masliah E, Rockenstein E, Veinbergs I (2000). Dopaminergic loss and inclusion body formation in alpha-synuclein mice: implications for neurodegenerative disorders. Science.

[CR26] Mcginty JF (2007). Co-localization of GABA with other neuroactive substances in the basal ganglia. Prog Brain Res.

[CR27] Mittal S, Bjornevik K, Im DS (2017). Beta2-adrenoreceptor is a regulator of the alpha-synuclein gene driving risk of Parkinson’s disease. Science.

[CR28] Miyazaki H, Oyama F, Inoue R (2014). Singular localization of sodium channel beta4 subunit in unmyelinated fibres and its role in the striatum. Nat Commun.

[CR29] Mori F, Tanji K, Yoshimoto M, Takahashi H, Wakabayashi K (2002). Immunohistochemical comparison of alpha- and beta-synuclein in adult rat central nervous system. Brain Res.

[CR30] Moulder KL, Mennerick S (2005). Reluctant vesicles contribute to the total readily releasable pool in glutamatergic hippocampal neurons. J Neurosci.

[CR31] Moulder KL, Jiang X, Taylor AA, Shin W, Gillis KD, Mennerick S (2007). Vesicle pool heterogeneity at hippocampal glutamate and GABA synapses. J Neurosci.

[CR32] Pavlou MAS, Colombo N, Fuertes-Alvarez S (2017). Expression of the parkinson’s disease-associated gene alpha-synuclein is regulated by the neuronal cell fate determinant TRIM32. Mol Neurobiol.

[CR33] Petersen K, Olesen OF, Mikkelsen JD (1999). Developmental expression of alpha-synuclein in rat hippocampus and cerebral cortex. Neuroscience.

[CR34] Polymeropoulos MH, Lavedan C, Leroy E (1997). Mutation in the alpha-synuclein gene identified in families with Parkinson’s disease. Science.

[CR35] Scherzer CR, Grass JA, Liao Z (2008). GATA transcription factors directly regulate the Parkinson’s disease-linked gene alpha-synuclein. Proc Natl Acad Sci USA.

[CR36] Scott D, Roy S (2012). Alpha-synuclein inhibits intersynaptic vesicle mobility and maintains recycling-pool homeostasis. J Neurosci.

[CR37] Singleton AB, Farrer M, Johnson J (2003). Alpha-synuclein locus triplication causes Parkinson’s disease. Science.

[CR38] Smith AD, Bolam JP (1990). The neural network of the basal ganglia as revealed by the study of synaptic connections of identified neurones. Trends Neurosci.

[CR39] Spillantini MG, Crowther RA, Jakes R, Hasegawa M, Goedert M (1998). Alpha-synuclein in filamentous inclusions of Lewy bodies from Parkinson’s disease and dementia with lewy bodies. Proc Natl Acad Sci USA.

[CR40] Stefanis L (2012). Alpha-synuclein in Parkinson’s disease. Cold Spring Harb Perspect Med.

[CR41] Sugiyama T, Osumi N, Katsuyama Y (2014). A novel cell migratory zone in the developing hippocampal formation. J Comp Neurol.

[CR42] Taguchi K, Watanabe Y, Tsujimura A (2014). Differential expression of alpha-synuclein in hippocampal neurons. PLoS One.

[CR43] Taguchi K, Watanabe Y, Tsujimura A, Tanaka M (2016). Brain region-dependent differential expression of alpha-synuclein. J Comp Neurol.

[CR44] Totterdell S, Meredith GE (2005). Localization of alpha-synuclein to identified fibers and synapses in the normal mouse brain. Neuroscience.

[CR45] Totterdell S, Hanger D, Meredith GE (2004). The ultrastructural distribution of alpha-synuclein-like protein in normal mouse brain. Brain Res.

[CR46] Utter AA, Basso MA (2008). The basal ganglia: an overview of circuits and function. Neurosci Biobehav Rev.

[CR47] van der Perren A, Toelen J, Casteels C (2015). Longitudinal follow-up and characterization of a robust rat model for Parkinson’s disease based on overexpression of alpha-synuclein with adeno-associated viral vectors. Neurobiol Aging.

[CR48] Vivacqua G, Casini A, Vaccaro R, Fornai F, Yu S, D’Este L (2011). Different sub-cellular localization of alpha-synuclein in the C57BL\6 J mouse’s central nervous system by two novel monoclonal antibodies. J Chem Neuroanat.

[CR49] Volpicelli-Daley LA, Luk KC, Patel TP (2011). Exogenous alpha-synuclein fibrils induce Lewy body pathology leading to synaptic dysfunction and neuron death. Neuron.

[CR50] Wilson CJ (1993). The generation of natural firing patterns in neostriatal neurons. Prog Brain Res.

[CR51] Withers GS, George JM, Banker GA, Clayton DF (1997). Delayed localization of synelfin (synuclein, NACP) to presynaptic terminals in cultured rat hippocampal neurons. Dev Brain Res.

[CR52] Yang YX, Latchman DS (2008). Nurr1 transcriptionally regulates the expression of alpha-synuclein. Neuroreport.

[CR53] Zarranz JJ, Alegre J, Gomez-Esteban JC (2004). The new mutation, E46 K, of alpha-synuclein causes Parkinson and Lewy body dementia. Ann Neurol.

